# Fetuin-A and Ghrelin Levels in Children with End Stage Renal Disease and the Effect of a Single Hemodialysis Session on Them

**DOI:** 10.3889/oamjms.2015.081

**Published:** 2015-07-17

**Authors:** Mohamed Gamal Shouman, Nagwa Abdallah Ismail, Ahmed Badr, Safaa Mohamed Abdelrahman, Shadia Ragab, Hebatallah Farouk

**Affiliations:** 1 *Department of Pediatrics, National Research Centre, Cairo, Egypt*; 2 *Department of Pediatrics, Faculty of Medicine, Cairo University, Cairo, Egypt*; 3 *Department of Clinical Pathology, National Research Centre, Cairo, Egypt*

**Keywords:** Fetuin-A, acyl ghrelin, hemodialysis, chronic renal failure, children

## Abstract

**BACKGROUND:**

Fetuin-A and ghrelin have been implicated in cardiovascular diseases and mortality among end stage renal disease patients. The exact mechanisms have not been fully elucidated. There is robust data supporting an association between ghrelin and various cardiovascular conditions, and some common processes such as inflammation, oxidative stress, and endoplasmic reticulum stress have been implicated.

**AIM:**

This study was conducted to assay serum fetuin-A and ghrelin in chronic renal failure pediatric patients and to study changes in their level that may occur after a single hemodialysis.

**MATERIAL AND METHODS:**

Forty nine pediatric patients suffering from ESRD on maintenance hemodialysis (HD), 20 patients with chronic renal failure (CRF) not on dialysis and 35 healthy subjects as control group were included. The mean age of the study population was 10.58 ± 3.94, 10.62 ± 3.24 and 10.61 ± 3.97 years respectively. Serum fetuin-A and plasma acyl ghrelin levels were measured by using ELISA method.

**RESULTS:**

The present study revealed that predialysis serum fetuin-A level was significantly increased in pediatric HD patients compared with the normal population, while ghrelin levels were significantly reduced. Furthermore, serum levels of fetuin-A decreased significantly after a single HD session.

**CONCLUSION:**

Our study concluded that fetuin-A and acyl ghrelin may play a role in inflammatory process among HD pediatric patients which may account for cardiovascular insults and mortality but their use as biochemical markers among ESRD pediatric patients have limitations due to wide fluctuations.

## Introduction

Hemodialysis (HD) patients experience a cardiovascular mortality of up to 20% per year, and vascular calcification is a strong independent risk factor of cardiovascular death [1, 2]. Fetuin-A (AHSG, a2-Heremans–Schimd glycoprotein) is an important inhibitor of vascular calcification [3, 4]. Fetuin uptake and secretion by proliferating and differentiating cells in the arterial wall is a protective mechanism against arterial calcification [5]. Circulating fetuin-A decreases in parallel with decline in renal function [6]. Reduced serum fetuin-A levels are associated with inflammation and increased cardiovascular mortality in hemodialysis [7].

Ghrelin, a peptide of 28 amino acids, was first reported by Kojima in rat and human stomachs in 1999 [8]. There is robust data supporting an association between ghrelin and various cardiovascular conditions, and some common processes such as inflammation, oxidative stress, and endoplasmic reticulum stress have been implicated, although the exact mechanisms have not been fully elucidated [9]. Ghrelin may have cardiovascular protective effect, including lowering of blood pressure, regulation of atherosclerosis, and protection from ischemia/reperfusion injury as well as improving the prognosis of myocardial infarction and heart failure. Some of these new functions of ghrelin may provide new potential therapeutic opportunities for ghrelin in cardiovascular medicine [9]. There is an urgent need for effective appetite-stimulatory therapies for chronic kidney disease (CKD) patients. Ghrelin is more potent than any other orexigenic factors as it rapidly enhances food consumption following injection in rodents [10] and humans [11]. Results of recent findings bolster the potential therapeutic application of ghrelin and its analogues as an appetite stimulating and anabolic strategy in uremia associated cachexia and other types of disease-associated cachexia. Ghrelin regulates metabolic balance and may improve the cachectic condition through IGF-dependent and IGF-independent pathways. Conflicting results of circulating ghrelin levels in CKD have been presented. Elevated plasma ghrelin levels were observed in adult dialysis patients than those of age-matched controls [12]. Szczepanska et al. reported that plasma ghrelin levels were similar in CKD children on dialysis compared with children on conservative treatment and healthy controls [13].

This study was primarily conducted to investigate changes in serum fetuin-A and plasma acyl ghrelin that may occur after a single hemodialysis in renal failure children and compare it to CRF and control children.

## Subjects and Methods

This cross-sectional study was carried out on 49 ESRD pediatric patients on maintenance hemodialysis and 20 patients with CRF not on dialysis recruited from the Pediatric Dialysis Unit, Cairo University Children Hospital. Thirty five healthy subjects were served as control group. The studied hemodialysis pediatric patients consisted of 22 (44.8%) females and 27(55.2%) males. The mean age of the study population was 10.58 ± 3.94 years for hemodialysis patients, 10.62 ± 3.24 for CRF patients and 10.61 ± 3.97 years for controls. The mean time of dialysis of these patients suffering from ESRD was 49.44 ± 33.25 months. Hemodialysis was performed thrice weekly for about 3 to 4 h per session at blood flow rates of 4-5 ml/kg/min using high flux polysulfone hollow-fiber filter. Exclusion criteria included active infection. The study was approved by the Ethical Committee of National Research Centre and informed consent was obtained in every case from their legal guardians.

Demographic data, anthropometric measurements (weight, height, BMI, mid-arm circumference, triceps skin fold thickness), primary renal disease, dialysis duration, vital signs, and routine laboratory investigations were obtained. Systolic (SBP) and diastolic blood pressure (DBP) was measured with a standard clinical sphygmomanometer. Body mass index was calculated as weight divided by height squared for each patient (kg/m^2^). Routine laboratory investigations included complete blood count (using Coulter counter), calcium, phosphorous, alkaline phosphatase, creatinine, BUN, Na, K.

For Serum fetuin-A and plasma acyl ghrelin levels assay, blood samples were collected before hemodialysis and also after hemodialysis. Fasting blood sample was collected from CRF patients and controls. All serum and plasma were frozen at -80°C. Serum fetuin-A levels in human subjects were measured using commercial enzyme-linked immunosorbent assays (ELISA, BioVendor Laboratory Medicine, Brno, Czech Republic), and plasma acyl ghrelin levels were measured by using ELISA method.

### Statistical Analysis

Standard computer program SPSS for Windows, release 13.0 (SPSS Inc., USA) was used for data entry and analysis. All numeric variables were expressed as mean ± standard error of mean (SE). Comparison of different variables in various groups was done using Student t test and Mann Whitney test for normal and nonparametric variables respectively. Pearson’s and Spearman’s correlation tests (r = correlation coefficient) were used for correlating normal and non-parametric variables respectively. For all tests, a probability (P) less than 0.05 (< 0.05) is considered significant.

## Results

The characteristics of the study population are shown in Table 1. The primary renal diseases leading to ESRD among HD pediatric children were urinary tract infection and obstructive uropathy (N = 23), glomerulonephritis (N = 7), congenital renal disease (N = 7), and unknown cause (N = 12). The differences between the mean of serum levels of fetuin-A and acyl ghrelin between HD patients compared with control population are shown in Table 2.

**Table 1:** Characteristics of study participants

Variables	Hemodialysis (No = 49 patients)	CRF (No = 20)	Controls (No = 35)	P-value
Age (years) mean ± SD	10.58 ± 3.94	10.62 ± 3.24	10.61 ± 3.97	>0.05
Sex (M/F ratio)	27/22	10-Oct	18/17	>0.05
Body mass index (kg/m^2^)	17.44 ± 2.88	16.65 ± 3.68	20.23 ± 2.93	<0.05
Duration of dialysis (years)	2.97 ± 1.78	-		
Systolic blood pressure (mm Hg)	118.36 ± 18.41	89.23 ± 5.8		
Diastolic blood pressure (mm Hg)	78.36 ± 12.72	58.46 ± 3.91		
Hypertension	30/49 (61.2%)	0		
Hb g/dl	10.44 ± 2.20	10.5 ± 1.57		
Hct %	31.06 ± 8.91	30.78 ± 3.94		
WBCs ×10^3^/μL	7.23 ± 2.57	7.4 ± 2.73		
Platelets ×10^3^/μL	209.05 ± 92.02	258.33 ± 75.78		
Ca (mg/dl)	8.7 ± 1.03	9.18 ± 1.28		
P (mg/dl)	5.27 ± 1.57	4.64 ± 0.82		
Alkaline phosphatase (IU/L)	585.15 ± 357.9	515.7 ± 323.2		
Albumin (mg/ml)	3.47 ± 0.32	3.67 ± 0.47		
Urea (mg/dl)	73.59 ± 30.55	52.31 ± 21.51		
Creatinine (mg/dl)	6.91 ± 2.2	2.58 ± 1.28		
Fetuin-A (μg/ml)	1093.9 ± 930.9	516.5 ± 54.1	449.1 ± 60.43	
Fetuin-A (post-dialysis)	503.67 ± 349.9			
Acylated Ghrelin (pg/ml)	127.28 ± 9.44	129.5 ± 3.5	140.21 ± 11.87	
Acylated Ghrelin (post-dialysis)	132.13 ± 6.02			
Kidney disease diagnosis				
Nephritis	7 (14.3%)	3 (15%)		
UTI and obstructive uropathy	23 (46.9%)	8 (40%)		
Congenital renal defects	7 (14.3%)	6 (30%)		
Unknown	12 (24.5%)	3 (15%)		

UTI: urinary tract infection

**Table 2:** Comparison of serum fetuin-A and plasma ghrelin between pediatric dialysis patients and controls

	N	Mean ± SD	Sig. (2-tailed)
Fetuin- A predialysis	49	1093.94 ± 930.79	
Control	35	449.06 ± 76.19	.000
Ghrelin predialysis	49	127.28 ± 9.44	
Control	35	140.21 ± 11.87	.000

The present study showed that HD patients have significantly increased serum fetuin-A levels and reduced plasma ghrelin levels as compared with the normal population. Furthermore, serum levels of fetuin-A decreased significantly during a single HD session while plasma ghrelin was insignificantly affected (Table 3). The serum fetuin-A and acyl ghrelin in CRF patients was not significantly affected as compared to control. The correlation between serum fetuin-A and plasma ghrelin and anthropometric measures for patients and controls revealed significant negative correlation between fetuin-A in patients and weight (p value = 0.03). There were also positive significant correlations between blood pressure (systolic and diastolic) and weight and height. The correlations between fetuin-A and acyl ghrelin and renal function parameters revealed no significant correlation between them and GFR among CRF group.

**Table 3:** Paired Samples Test for fetuin-A and ghrelin pre and postdialysis

Paired Samples Test		Paired Differences					t	df	Sig. (2-tailed)

		Mean	Std. Deviation	Std. Mean	Error	95% Confidence Interval of the Difference			

					Lower	Upper			
Pair 1	Fetuin-A predialysis - Fetuin-A postdialysis	559.16	814.77	235.20	41.48	1076.85	2.37	11	.037
Pair 2	Ghrelin predialysis - Ghrelin postdialysis	-4.01	12.55	2.29	-8.70	.67	-1.75	29	.090

## Discussion

Pediatric patients suffering from ESRD, whether on regular dialysis or on conservative therapy, have a large burden of cardiovascular diseases. One of the important risk factor is inflammation. The results of the present study showed that patients with renal diseases whether on regular HD or on conservative therapy display various degrees of changes in some biochemical parameters.

In adult studies, serum fetuin-A levels were significantly lower in hemodialysis patients as compared to CRF patients and controls [14, 15]. In pediatric patients, there was a controversy about the serum level of fetuin-A on regular hemodialysis. In this study, serum fetuin-A levels were significantly higher in hemodialysis pediatric patients as compared to CRF patients and controls while no significant difference was found between CRF patients and controls. Similar to our study, ZiÃ^3^Å et al study revealed that the serum fetuin-A in CRF patients was not significantly affected as compared to control [16]. Schaible et al study revealed that fetuin-A levels are clearly reduced in children on dialysis but not in those with moderate CKD and after transplantation [17]. Fetuin-A was lower in dialyzed and renal transplanted children compared with healthy controls [18]. Similar to our study, Shroff et al study on 16 children stated that physiological inhibitors of calcification, fetuin-A, osteoprotegerin (OPG) and under carboxylated-matrix Gla protein (uc-MGP) may play a role in preventing the development and progression of ectopic calcification, fetuin-A were higher in dialysis patients than controls. Fetuin-A showed an inverse correlation with dialysis vintage, time-averaged serum phosphate and hs-CRP [19]. Few studies in adult revealed that fetuin-A levels were significantly lower in the CKD group compared to the controls and no correlation between GFR and serum fetuin-A [7, 20, 21]. In Kayser et al study, serum fetuin-A concentrations were found to be decreased in all adult CKD patients but stage 1 CKD [22]. Cottone et al studies stated that plasma levels of fetuin-A were independently and directly associated with eGFR in CKD patients, showing a progressive and significant decrease related to decreasing eGFR [6, 23].

Regarding in vivo circulating modulators of calcification, fetuin-A is a circulating calcium-regulatory protein inhibiting Ca=PO_4_ precipitation and it seems to be involved in both inflammation and vascular calcification processes. Actually, it is known that fetuin-A is down regulated during systemic inflammation as a negative acute phase protein [24]. In our study, serum fetuin-A level was significantly reduced after a single HD session. Ciaccio et al study concluded that the significant decrease of fetuin-A levels after a single HD session is consistent with the hypothesis of HD-induced inflammation [24]. Dusilova et al study found that serum concentrations of fetuin-A in hemodialysis (HD) patients are low. This decrease is conventionally explained by malnutrition and inflammation seen often in HD patients. Serum fetuin-A decreased significantly during low-flux hemodialysis. The hemodialysis procedure itself is accompanied by a significant decrease of serum fetuin A, which may at least theoretically contribute to low serum fetuin-A concentrations generally observed in HD patients. This decrease is not related to bone mineral metabolism and/or inflammation markers. Because molecular weight of serum fetuin-A indicates no permeability of dialysis membrane, other yet unknown mechanisms are involved in consumption of this molecule during HD [25]. Fetuin-A levels were independent of age, pubertal stage, and gender. Fetuin-A correlated significantly to systolic and diastolic blood pressure, Fetuin-A levels were higher in obese children with Non Alcoholic Fatty Liver Disease, and were related to insulin resistance and to features of the MetS in both cross-sectional and longitudinal analyses [26]. Safranek et al study in adult showed that low levels of fetuin-A are associated with malnutrition, inflammation, decreased bone mass density, low-turnover bone and use of high calcium concentration dialysate. Hemodialysis procedure (HD) has been shown to decrease fetuin-A levels by 20%, probably due to HD-induced inflammation or acute changes in calcium metabolism. Also Safranek et al study in adult concluded that standard bicarbonate HD with polysulfone dialyser and ultrapure dialysate induces only minor changes in fetuin-A [27].

Small studies of children with CKD stages 2 to 4 or on dialysis therapy have shown rates of hyperinsulinemia as high as 33% and rates of abnormal insulin resistance (measured by increased Homeostasis Model Assessment for Insulin Resistance) in up to 16% of patients, indicating that abnormalities in insulin and glucose metabolism may be present earlier [28, 29].

There are several limitations to our study. First, this is a relatively small sample size. Second, the studies in pediatric age are few with controversial results. Third, there is considerable intrapatient variation fetuin-A. Tsirpanlis et al study and LaClair et al study followed patients for months; measuring weekly values; found variability in measured fetuin-A levels [30, 31]. LaClair et al study also measured four potential inflammatory biomarkers (CRP, IL-6, fetuin-A, albumin), all of which have limited specificity for inflammation [31].

Acylated active form of ghrelin mediates appetite regulation [32]. One major effect of ghrelin is the increase of food intake with a subsequent increase of body weight, fat accumulation and increase in carbohydrate oxidation [33, 34]. Pediatric patients with ESRD often suffer from insufficient weight gain and growth retardation due to low appetite and low calorie intake. This uremic malnutrition is associated with high morbidity, poor quality of life and increased mortality rates. The ghrelin is now under trial in animals and human to be used as treatment to stimulate appetite in ESRD patients [35, 36].

In our study, plasma acyl ghrelin levels were significantly reduced in pediatric hemodialysis patients as compared to CRF patients on conservative treatment and controls. This may explain the loss of appetite among hemodialysis patients. Wynne et al. study tested the effects of subcutaneous ghrelin administration in CKD patients with mild to moderate malnutrition with improvement energy intake [37]. In comparison to other studies, there was conflicting results as regard plasma level of total ghrelin and acylated active form. Arbeiter et al. study [38] in pediatrics revealed that total ghrelin is significantly increased in CRF children which is similar to few studies in uremic children and adults [32, 39, 40, 41], while acyl ghrelin did not differ between hemodialysis, CRF, and control groups [41, 42]. Similar to our study, Montazerifar et al and Amanda de Faria Barros et al studies in adult revealed that acyl ghrelin concentration was significantly declined in HD patients when compared to healthy controls [43, 44]. Eftekhari et al study in children showed insignificant elevated total ghrelin [45]. In our study and others, the plasma acyl ghrelin is not significantly affected in spite of its clearance with hemodialysis [32, 40]. There were few limitations to our study on ghrelin. First, small sample size. Second, little is known about the regulation of ghrelin in children with CRF and during dialysis treatment since all studies included only few paediatric patients. Third, the ghrelin form assessed differed in different studies. In this study, acylated active ghrelin was assessed. Yoshimoto et al study revealed that the plasma level of the acylated active form of ghrelin is not raised in uremic patients, but instead the level of the non-acylated ghrelin [32]. Büscher et al study revealed that acyl ghrelin did not differ between groups; in children with CKD, children undergoing hemodialysis or peritoneal dialysis, renal transplant recipients, and healthy controls [42]. In our study, no correlation was found between GFR and acyl ghrelin which is similar to Naufel et al study [41], while in Arbeiter et al. study [38], and Naufelet al study [41] a negative correlation between glomerular filtration rate and total ghrelin was observed in CRF and transplant recipients. Fourth, despite animal models showing reduced clearance of ghrelin in rodent models of renal failure, conflicting results (i.e., increase, decrease, or no change) of circulating ghrelin concentrations in CKD patients have been reported. Szczepańska et al study found that plasma ghrelin levels were similar in children on APD and children on conservative treatment compared to healthy controls [13]. In another study, plasma ghrelin levels were markedly increased in HD and PD patients compared to healthy controls, while there was no difference between HD and PD patients [12]. Iglesias et al. study showed that patients undergoing HD have similar ghrelin concentrations in comparison with the control group. However, PD patients exhibited baseline ghrelin concentrations significantly lower than those found in patients on conservative management [46]. The available literature on plasma level of ghrelin in dialysis patients is not consistent, even within the same age range [46-48]. Plasma ghrelin levels increase before meals (rather than remaining low or stable), increase food intake (rather than increasing satiety), and decrease postprandial (rather than increasing) [49]. Due to this regulation, studies that include only a single fasting level may miss significant changes in 24-hour exposure that are due to alterations in postprandial suppression [50].

Our study concluded that fetuin-A and acyl ghrelin may play a role in inflammatory process among HD pediatric patients which may account for cardiovascular insults and mortality but their use as biochemical markers among ESRD pediatric patients have limitations due to wide fluctuations.

Future studies in pediatric ESRD patients’ weather on conservative therapy or on hemodialysis are needed to learn more about the role of fetuin-A and ghrelin in diseased children.
